# Long-term BPV is an Independent Risk Factor for Renal Prognosis in Hypertensive Patients — a Post-hoc Analysis of the SPRINT Study

**DOI:** 10.7150/ijms.111843

**Published:** 2025-04-22

**Authors:** Yuyi Ruan, Yutong Chen, Naya Huang, Dan Wang, Yuzhu Xu, Jinjin Fan, Wei Chen, Xin Wang

**Affiliations:** 1 Department of Nephrology, The First Affiliated Hospital, Sun Yat-sen University, Guangzhou, 510080, China.; 2 NHC Key Laboratory of Clinical Nephrology (Sun Yat-Sen University) and Guangdong Provincial Key Laboratory of Nephrology, Guangzhou, 510080, China.

**Keywords:** blood pressure, blood pressure variability, hypertension, renal insufficiency, renin angiotensin system

## Abstract

**Background:** Long-term blood pressure variability (BPV) reflects fluctuations in BP over time, which may indicate instability in precise blood pressure control. We conducted a post hoc analysis of the data from the SPRINT (Systolic Blood Pressure Intervention Trial) to assess the effect and associated variables of BPV on the renal prognosis of patients with hypertension.

**Methods:** Excluding patients with CKD, the systolic blood pressure (SBP) at the 1^st^, 6^th^, and 12^th^ follow-up months were employed to calculate the SBP coefficient of variation (CV) which represented BPV. Patients were divided into four groups based on the quartiles of BPV, namely Q1 to Q4.

**Results:** Group Q4 patients had higher baseline SBP. Multiple regression identified age, sex, treatment, current smoker, SBP, diastolic blood pressure (DBP), renin-angiotensin-system inhibitors (RASi), β-receptor antagonists, calcium channel blockers (CCBs), and other medications use were factors associated with BPV. The survival analysis showed that group Q4 had significantly more renal outcome events, and BPV was independently associated with the risk of renal outcome events (HR = 1.38, 95% CI: 1.23 - 1.54, *P* < 0.001). There was a direct correlation between the BPV and risk of renal outcomes when BPV exceeded 0.037. In addition, the RASi preference group reported a significantly higher incidence of renal outcome events compared to the non-preference group (log-rank test χ² = 6.218, *P* = 0.013) and exhibited a tendency towards higher BPV.

**Conclusions:** High BPV is an independent risk factor for renal outcome events in hypertensive aging patients. The preference of RASi use can increase renal outcome events, but is not related to the rise in BPV. These findings suggest that in elderly hypertensive patients with elevated BPV, the potential risks of RASi-associated renal outcomes may outweigh its established benefits, necessitating cautious consideration of alternative antihypertensive strategies.

## Introduction

Hypertension is a common disease of the elderly. During the period from 1990 to 2019, the number of hypertensive patients aged between 30 and 79 years has doubled 1. Despite the implementation of various health management policies, the global status of hypertension control is still unsatisfactory 2. Uncontrolled hypertension can cause vascular damage that contributes to poor patient prognosis by damaging multiple target organs, including the heart, brain and kidney 3. Intensive blood pressure (BP) lowering has widely recognized benefits for the heart and brain, but its effects on the kidneys remain controversial 4.

In addition to blood pressure levels, blood pressure variability (BPV) has not been adequately addressed by clinicians as an important indicator of blood pressure control. BPV corresponds to the fluctuations in BP during a certain period, which are often indicated by the coefficient of variation (CV), calculated as dividing the standard deviation of the ambulatory BP by its mean. These changes are mainly related to hemodynamic changes, vascular sclerosis and activation of the renin-angiotensin-aldosterone system (RAAS), and it is therefore interesting to know whether different mechanisms of antihypertensive drugs affect BPV or renal prognosis 5. Based on observation time, BPV can be classified into three groups; per-beat BPV, short-term BPV (24-hour BPV), and long-term BPV (visit-to-visit BPV) 6. Several clinical studies have shown that elevated BPV, regardless of BP level, especially long-term and short-term variations, was an important risk factor for cardiovascular prognosis 7-9. Elevated short-term BPV has also been shown to increase renal outcomes 10,11. However, research into the renal prognosis of hypertensive patients using long-term BPV is currently very limited. Long-term BPV is more indicative of BP that has been relatively controlled and stabilized. To investigate the impact of long-term BPV on renal prognosis in elderly hypertensive patients and to provide more support for the clinical optimization of antihypertensive treatment regimens, we included non-CKD and non-DM hypertensive populations from the SPRINT study and reanalyzed the data.

## Methods

### Study population

The original database of the SPRINT study was acquired from the NHLBI's BioLINCC application. After excluding individuals with chronic kidney disease (CKD) [estimated glomerular filtration rate (eGFR)<60 mL/(min·1.73m^2^)] 4 and those who lacked follow-up information on their systolic blood pressure (SBP) within one year, we added 5849 hypertensive cases without diabetes mellitus (DM) or CKD.

### Definitions

1. Blood pressure variability (BPV) is represented by the coefficient of variation (CV) of SBP calculated from data obtained at the 1^st^, 6^th^, and 12^th^ follow-up months. It is determined by the ratio of the standard deviation of the SBP to the mean obtained at these three follow-up time points (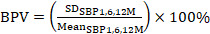).

2. CKD: eGFR < 60 mL/(min·1.73m^2^).

3. Renal outcome: eGFR decrease of 30% or more, or eGFR < 60 mL/(min·1.73m^2^) 4.

4. Acute kidney injury (AKI): defined regarding the SPRINT study, acute kidney injury or acute renal failure in a discharge diagnosis or emergency case, or AKI as determined by the safety officer 4.

### Group

Patients with hypertension, but without CKD, who participated in the SPRINT study were classified into 4 groups based on the quartiles of patients' BPV in the first year: Q1 (≤ 0.0432), Q2 (0.0432 - 0.0711), Q3 (0.0711 - 0.1072), and Q4 (> 0.1072). Each antihypertensive drug type was divided into two categories, namely the presence and non-presence groups, according to the presence or absence of drug use during the 1^st^, 6^th^, and 12^th^-month follow-up visits (drug use at those times qualified for the presence group while drug non-use was classified as the non-presence group).

### Observations

Renal outcome events, AKI adverse events, SBP, diastolic blood pressure (DBP), eGFR, and BPV.

### Statistical methods

SPSS 26.0 software and the R programming language were used for statistical analysis and graphing. Measurements were expressed as median and first and third quartiles, and counts were expressed as frequencies and percentages. Comparison of measurements among the 4 groups was performed by Kruskal-Wallis H test, and comparison of counts was performed by chi-square test. Kaplan-Meier (KM) analysis was performed with renal outcome events and AKI adverse events as dependent variables and quartile grouping of BPV as independent variables, and variables with *P* < 0.05 were included in Cox regression analysis. Cox regression analysis was used to analyze the risk factors associated with renal outcomes in patients. Restricted cubic spline (RCS) analysis was utilized to describe the nonlinear relationship between the BPV and the occurrence of renal outcome events. The preference of different medication types was unbalanced with statistically significant differences in major baseline characteristics and was balanced after 1:1 propensity scores match (PSM) by combining sex, age, treatment, cardiovascular disease history, current smoker, Framingham 10year cardiovascular disease risk score, Framingham 10year cardiovascular disease risk score ≥ 15%, SBP, DBP, heart rate, eGFR, urinary microalbumin, urine albumin creatinine ratio, total cholesterol, triglycerides, glucose, BMI and baseline drugs use. It was matched with a caliper value of 0.02 to make each matched factor balanced and comparable. KM analysis was performed with renal outcome events as the dependent variable and subgroups of antihypertensive medication preference as the independent variable. Mediation analysis was performed with RASi preference as the independent variable, BPV as the mediator variable, and renal prognosis as the dependent variable.

## Results

### Baseline information

**Figure 1:**
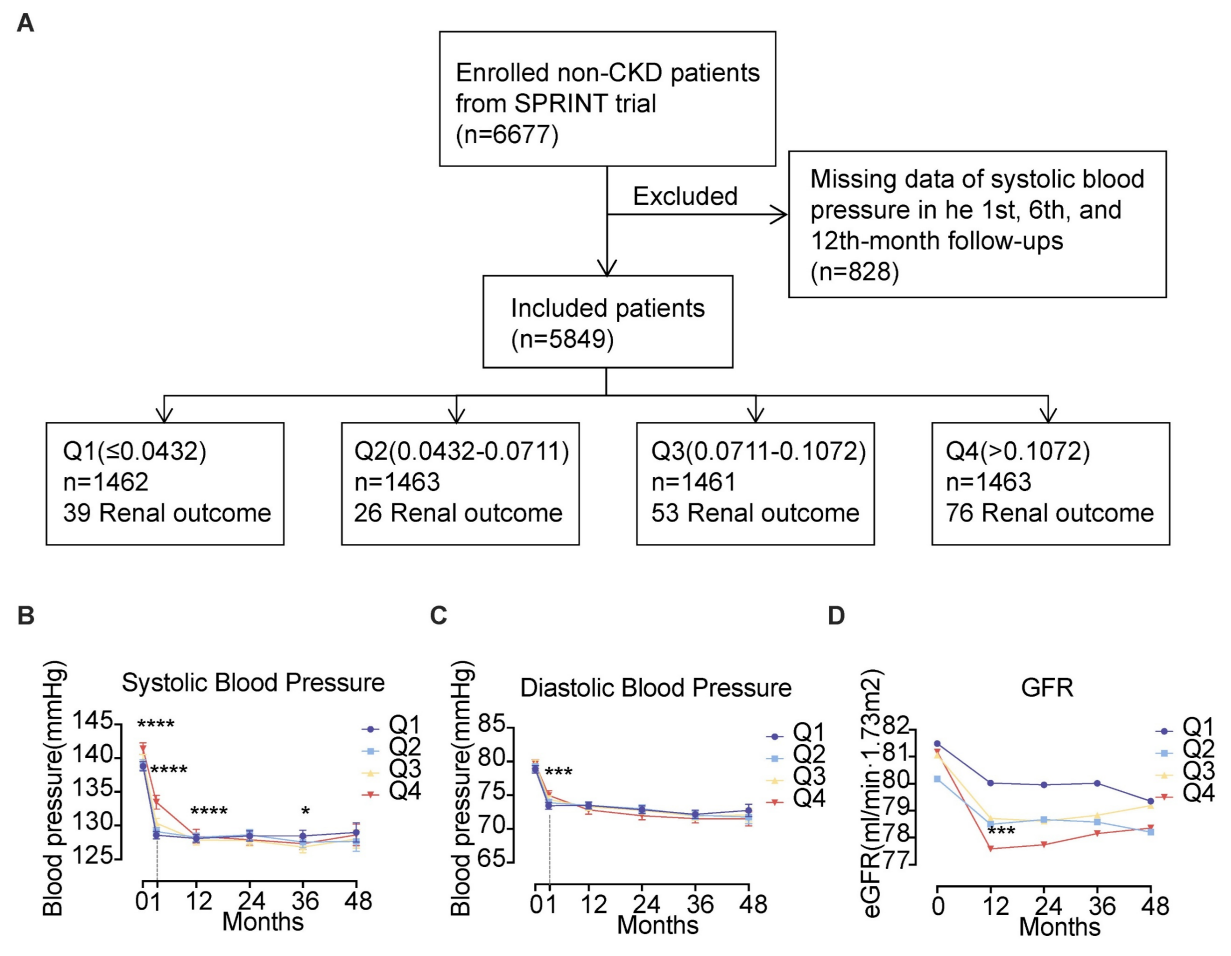
Follow-up of 5849 cases of hypertension. (**A**) Enrollment flow chart for the analysis of 5849 cases of hypertension. (**B**) Systolic blood pressure, (**C**) Diastolic blood pressure and (**D**) eGFR levels during follow-up. Abbreviations: eGFR, estimated glomerular filtration rate; Q1 to Q4, lowest to highest quartile.

This study finally included 5849 cases, and the inclusion process was shown in **Fig. 1A**. A total of 5,849 cases were enrolled, which comprised 1,925 females (32.9%) and 3,924 males (67.1%), with the median age being 65 years. Standard treatment (a SBP target of less than 140 mmHg) was provided to 50.1% of the cases, whereas 49.9% received intensive treatment (a SBP target of less than 120 mmHg). At baseline, the median SBP was 139 mmHg, and the median DBP was 79 mmHg. The baseline eGFR of the enrolled cases was all above 60 mL/(min·1.73m^2^), with a median urinary microalbumin of 10 mg/dL and a median urinary albumin to creatinine ratio of 8.62 mg/g. The enrolled cases had a BPV ranging from 0 to 0.4786. Participants were stratified into quartiles based on BPV measurements. The four groups were named Q1 (≤ 0.0432) with 1462 cases, Q2 (0.0432 - 0.0711) with 1463 cases, Q3 (0.0711 - 0.1072) with 1461 cases, and Q4 (> 0.1072) with 1463 cases. The proportion of patients receiving intensive treatment in each of the groups was 46.0%, 48.7%, 51.5%, and 53.3%, respectively. The percentage of smokers among the groups was as follows: 13.3%, 13.3%, 15.3%, and 18.3%, respectively. In each of the respective groups, the percentage of patients with a history of cardiovascular disease was: 16.6%, 17.4%, 19.5% and 20.0%. In comparison to the Q1, Q2, and Q3 groups, patients in the Q4 group had higher baseline systolic blood pressure (140 mmHg, Kruskal-Wallis H = 20.35, *P* < 0.001), greater urinary microalbumin (11 mg/dL, Kruskal-Wallis H = 13.97, *P* = 0.003) and higher urinary albumin to creatinine ratio (9.47 mg/g, Kruskal-Wallis H = 29.35, *P* < 0.001) (Refer to **Table 1** for further details).

### Risk factors associated with BPV

Multiple linear regression analysis identified several independent predictors of BPV, which were age (0.005, 95% CI: 0 - 0.009), sex (-0.181, 95% CI: -0.244 - 0.118), treatment (0.093, 95% CI: 0.041 - 0.146), current smoker (0.186, 95% CI: 0.092 - 0.279), baseline SBP (0.003, 95% CI: 0.000 - 0.005), baseline DBP (0. 004, 95% CI: 0.000 - 0.007), renin-angiotensin-system inhibitors (RASi) (0.109, 95% CI: 0.053 - 0.166), β-receptor antagonists (0.166, 95% CI: 0.107 - 0. 226), calcium channel blockers (CCBs) (-0.113, 95% CI: -0.167 - 0.059) and other medications (0.137, 95% CI: 0.043 - 0.231), as presented in **Table 2**.

### Follow-up BP and renal function in different groups of patients

During the 48-month follow-up, the Q4 group maintained significantly higher SBP levels at baseline and the first month of follow-up. However, no significant differences in SBP and DBP were observed between the different groups at other time points. All groups exhibited progressive eGFR declines, though no patient met the renal endpoint criteria. The Q4 group demonstrated the most pronounced eGFR reduction, showing statistically significant differences compared to Q1 at month 12 (P < 0.001). Inter-group comparisons revealed progressive eGFR declines across ascending BPV quartiles (Q1-Q4, all P < 0.001). See **Fig. 1B-D**.

### The influence of BPV on renal prognosis in hypertensive patients

**Figure 2:**
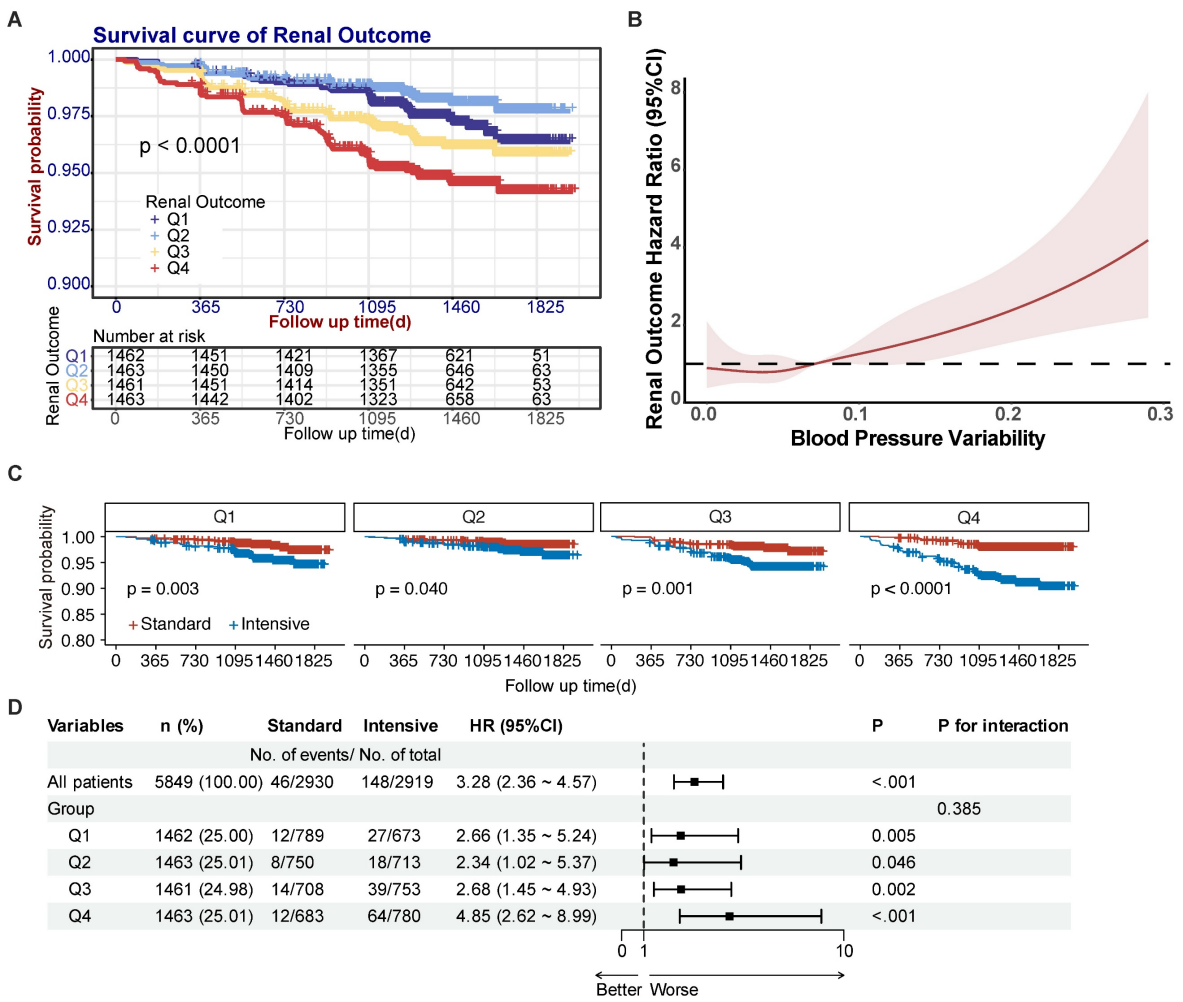
Effect of BPV on renal prognosis in hypertensive patients. (**A**) Survival curves for renal outcome events according to BPV quartiles. (**B**) Spline plots for risk of renal outcome events over the range of BPV. Curves represent hazard ratios (solid dark color line) and 95% CI (light color lines) based on restricted cubic splines analysis. (**C**) Survival curves for renal outcome events according to intensive treatment under BPV groups. (**D**) Forest plot for interaction effect between BPV and intensive treatment. Abbreviations: BPV, blood pressure variability; Q1 to Q4, lowest to highest quartile.

Kaplan-Meier analysis demonstrated significant renal outcome disparities across BPV quartiles (*P* < 0.001) (**Fig. 2A**). The Q2 group demonstrated the lowest incidence of renal outcome events (26 cases) while the Q4 group had the highest (76 cases). After accounting for confounding variables (Model 1: age, sex, treatment and current smoker; Model 2: Model 1 + SBP, DBP and urine microalbumin; Model 3: Model 2 + RASi, β-receptor antagonists, CCBs, and other medications), the BPV was found to be significantly associated with renal outcomes in hypertensive patients, with *P* value of 0.001. Additionally, event risk was found to be independently associated with an HR of 1.38 (95% CI: 1.23 - 1.54, *P* < 0.001) (refer to **Table 3**). In Model 3, the hazard ratio for renal outcomes per standard deviation increase in the BPV was 1.28 (95% CI: 1.13 - 1.44, *P* < 0.001). The hazard ratios for the Q1, Q3 and Q4 groups were subsequently adjusted using the Q2 group (which had the most promising prognosis) as a control, resulting in ratios of 1.68 (95% CI: 1.01 - 2.78, *P*= 0.044), 2.02 (95% CI: 1.25 - 3.26, *P* = 0.004), and 2.53 (95% CI: 1.60 - 3.99, *P* < 0.001). Patients of Q2 group demonstrated the most favorable prognosis compared to those in the Q1 group. These findings suggest a non-linear relationship between BPV and renal risk. Consequently, we conducted an additional analysis with restricted cubic splines (RCS) to visualize the relationship between BPV and renal outcomes. RCS analysis with 4 knots calculation result determined an inflection point of 0.037, indicating that the risk of renal outcomes in patients was least when the BPV was less than 0.037. Moreover, there was a direct correlation between BPV and the risk of renal outcomes above 0.037, as illustrated in **Fig. 2B**.

KM survival analysis with AKI adverse events as the endpoint revealed that only 9 AKI events occurred in the Q2 group and 16 AKI events occurred in both the Q3 and Q4 groups, but there was no significant difference in the incidence of AKI adverse events between the 4 groups (*P* = 0.268). The results of Cox analysis showed that after correction for confounders (Model 1: age, sex, treatment, current smoker, Model 2: Model 1 + SBP, DBP and urine microalbumin, Model 3: Model 2 + RASi, β-receptor antagonists, CCBs and other drugs), there was also no statistically significant difference between BPV and the risk of AKI in hypertensive patients. There was also no statistical difference in the risk of AKI adverse events (refer to **Table 4**).

### Interaction of BPV and intensive treatment on renal prognosis

SPRINT trial focused on multiple outcomes of patients from intensive and standard treatment groups. Since intensive treatment is a key factor in SPRINT trial, exploring the interaction between BPV and intensive treatment is important to investigate. Subgroup analyses stratified by BPV quartiles demonstrated all intensive treatment groups under Q1-Q4 groups had significant higher incidence of renal outcome events (**Fig. 2C**), which was consistent with result of intensive treatment group having a higher incidence of renal outcome events in SPRINT trial. Across quartiles, intensive therapy was associated with progressively increasing hazard ratios (Q1: 2.66; Q2: 2.34; Q3: 2.68; Q4: 4.85) relative to standard treatment (**Fig. 2D**). However, no significant interaction was observed between BPV and treatment intensity (P=0.385), indicating independent rather than synergistic effects (**Fig. 2D**).

### Effect of antihypertensive drug type on renal prognosis and BPV

**Figure 3:**
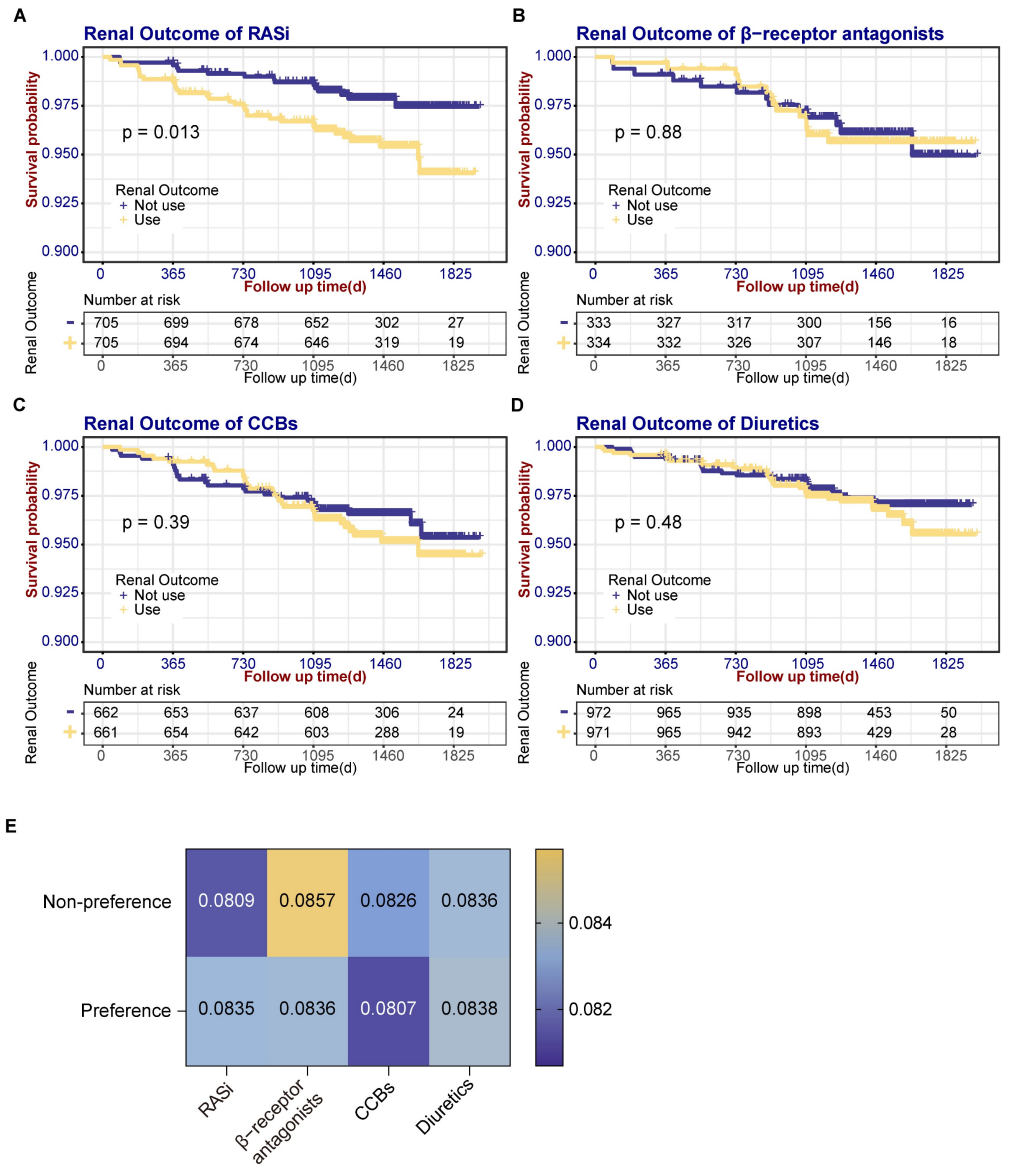
Renal outcome events survival curves according to antihypertensive drug types and association of BPV with antihypertensive drug types. (**A**) Renal outcome event survival curves according to the use of RASi. (**B**) Renal outcome event survival curves according to the use of β-receptor antagonists. (**C**) Renal outcome event survival curves according to the use of CCBs. (**D**) Survival curves for renal outcomes according to use of diuretics. (**E**) Comparison of BPV between medication preference and non-preference groups. CCBs, calcium channel blockers; RASi, renin-angiotensin-system inhibitors.

Given that BP in hypertensive patients is predominantly controlled by antihypertensive medications, and our analysis identified various antihypertensive drug classes as risk factors for BPV, we investigated whether these medications could mediate the relationship between BPV and renal outcomes. Participants were classified by predominant antihypertensive class during follow-up, those taking medication at the 1^st^, 6^th^ and 12^th^-month follow-ups were referred to as the 'preference group' and the others as the 'non-preference group'. Baseline data such as age, sex, treatment history of cardiovascular disease, current smoking status, as well as eGFR and microalbuminuria, were matched with PSM for both groups. Baseline characteristics of medications preference are shown in **Table S1-4**. SBP follow-up showed RASi, CCB and diuretic groups had similar patterns in SBP control with significantly higher SBP in 1st month in preference groups (**Fig. S1A**). In GFR follow-up, only CCB preference group had a trend of higher GFR before 24 months (**Fig. S1B**). A KM analysis of renal prognosis was then conducted. There was a significant increase in renal outcome events in the RASi preference group (32 events) compared with non-preference group (15 events) (Log-rank test χ² = 6.218, *P* = 0.013). In contrast, no increase in renal outcome events was observed in the β-receptor antagonists (Log-rank test χ² = 0.024, *P* = 0.876), CCBs (Log-rank test χ² = 0.734, *P* = 0.391), and diuretics (Log-rank test χ² = 0.491, *P* = 0.484) preference groups (refer to **Fig. 3A-D**). Separate BPV was determined for the matched subgroups of each type of antihypertensive medication. The analysis showed a higher BPV in the RASi preference group (0.0809 *vs.* 0.0835, *P* = 0.788), although the difference was not statistically significant. There are no significant differences in BPV among the β-receptor antagonists (0.0857 *vs.* 0.0836, *P* = 0.551), CCBs (0.0826 *vs.* 0.0807, *P* = 0.542) and diuretics (0.0836 *vs.* 0.0838, *P* = 0.275) preference group compared to the non-preference group, as shown in **Fig. 3E**.

### Mediation analysis of RASi-mediated BPV on renal prognosis

To delineate whether RASi mediated BPV on renal prognosis, we set RASi preference as the independent variable, BPV as the mediator variable, and renal prognosis as the dependent variable in the mediation model. Mediation analysis had two important parameters: average causal mediation effects (ACME) which reflected the indirect effect of RASi on renal prognosis through the pathway of BPV in this mediation model, and average direct effects (ADE) reflected the direct effect of RASi on renal prognosis. In our model, mediation analysis result showed ACME was 0.0007 (95% CI: -0.001 - 0.00, *P* = 0.388), ADE was 0.0232 (95% CI: 0.005 - 0.04, *P* = 0.018), and the proportion of mediation is 2.906% (*P* = 0.400) (**Fig. S2**). These results suggest that RASi preference affected renal prognosis mainly through the direct pathway, and not through the mediated pathway of BPV.

## Discussion

By a post hoc analysis of the SPRINT data in a population consisting solely of individuals with hypertension, we discovered that an elevation in BPV represented an independent risk factor for renal outcomes. Moreover, we noticed that the relationship between BPV and renal outcomes was non-linear and the lowest frequency of renal outcome events occurred when BPV was 0.037. The preference use of RASi was shown to significantly increase the incidence of renal outcomes but this was not mediated by the augmentation in long-term BPV.

As an indicator of long-term BP control homeostasis, BPV closely linked to blood vessel wall elasticity as evident from previous research 12. Our findings align with prior studies showing that age has a significant impact on BPV. Past research confirms that age-related progression towards vascular stiffening and resulting decreased wall elasticity is more pronounced in hypertensive individuals. DM and CKD are also common factors that promote the development of vascular sclerosis. Therefore, to investigate the long-term impact of BPV on renal prognosis, it is necessary to exclude age, DM, and CKD as risk factors to reduce bias.

Some studies have shown that an increase in BPV significantly increases the incidence of cardiovascular events in patients 13,14, but a post hoc analysis of the SPRINT study showed no correlation between the long-term BPV and both lethal and nonlethal cardiovascular composite endpoints 15. Our study, also looking at the BPV in SPRINT, found that it was associated with the occurrence of renal endpoints, suggesting that the kidney may demonstrate greater susceptibility to BPV-mediated damage compared to the cardiovascular system. A study in a Japanese population showed that long-term BPV was highly correlated with new-onset CKD and new-onset DM in a population with non-hypertensive at baseline. The results of this study excluded the effect of hypertension, suggesting that BP variability within the normotensive range remains an influential factor in renal prognosis 8. The CSPPT study showed that long-term BPV in Chinese hypertensive patients without a history of cardiovascular disease (CVD) and CKD increased the risk of CKD 16, which is in agreement with our findings. However, the hypertensive population of this study did not exclude DM patients and did not have multiple follow-ups on blood glucose, which is an important cause of promoting vascular sclerosis, so the findings of this study may have confounded the effect of hyperglycemia on the vascular and BPV 17,18. Of course, different levels of BP control in different studies, heterogeneity of study subjects, and different definitions of BPV used may also lead to differences in the results, so it is urgent to standardize the definition of BPV in the future.

AKI is a relatively common complication in the process of achieving hypertension control. Regardless of whether it is combined with CKD or not, the occurrence of AKI can lead to irreversible damage to renal function in patients 19. Therefore, we further explored the relationship between the BPV over a long period and adverse events of AKI. Our study showed that although the long-term BPV increased the occurrence of renal endpoint events, it was not significantly associated with AKI adverse events. Of course, we also noted that both the number of AKI adverse events and the number of renal end-point events were the lowest in the Q2 group and the highest in the Q4 group, whereas the involvement of AKI in the occurrence of renal end-point events could not be completely ruled out in the current study because of the small number of overall AKI cases. Given that there have been no studies on the correlation between long-term BPV and AKI, the possibility of a correlation should be further explored in a larger sample study at a later stage.

The most crucial method of protecting target organs in the treatment of hypertension now is strict BP control with various antihypertensive medications 20,21. The results of the SPRINT study strongly confirmed the cardiovascular and cerebrovascular protective effects of intensive antihypertensive therapy, which improved cardiovascular events and all-cause mortality. However, the effect of intensive treatment on the kidneys remains controversial. The rate of renal outcome events was significantly higher in patients in the intensive treatment group for non-CKD in the SPRINT study than in the standard treatment group (HR: 3.49; 95% CI: 2.44 to 5.10; *P* < 0.001). The investigators suggested that it might be related to reasons such as large blood pressure drops and more use of diuretics and RASi 4.

Our analysis revealed elevated renal outcome risk in the RASi preference group, contrasting with neutral outcomes observed in β-receptor antagonists, CCBs, or diuretics preference groups. While RASi remains the guideline-recommended choice for hypertensive CKD patients due to its urinary albumin-lowering effect 21, this renal protection may be counterbalanced by its hemodynamic impacts. Mechanistically, RASi reduces glomerular filtration pressure through preferential efferent arteriole dilation, which may accelerate renal function deterioration in advanced progressive CKD patients with critical renal artery stenosis 22, as evidenced by the STOP-ACEi trial showing renal function stabilization after ACEi withdrawal 23. Trials claiming renoprotection independent of BP effects were methodologically constrained by inconsistent BP control between groups 24,25. For instance, the seminal benazepril trial reported significantly lower BP in the intervention arm throughout follow-up, confounding the interpretation of its antiproteinuric effects 24. Similarly, a ramipril study showing reduced proteinuria in mild CKD patients simultaneously demonstrated greater DBP reductions in the treatment group, without significant GFR preservation 25. These limitations underscore the need for trial designs that rigorously dissociate BP-lowering effects from putative nephroprotective mechanisms.

Notably, emerging evidence suggests CCBs may provide complementary benefits through BPV reduction 26-28. Long-acting CCBs demonstrate renal protection via sustained BP control, particularly effective in non-dipping patients requiring nocturnal hypertension management 29, while short-acting formulations might exert pleiotropic effects through antioxidant and endothelial pathways. This highlights the need for personalized antihypertensive selection in elderly patients with elevated BPV.

There are some limitations in this study. The SPRINT study was terminated early due to the significant decrease in eGFR in some patients, so the total number of both renal events and AKI adverse events that occurred was small, which may have some impact on the significance of the results. The small number of AKI events (n = 42) may limit the statistical power of our analysis, and future studies with larger sample sizes are warranted. Because of the complexity of comorbid medications in hypertensive patients, this part of the study could only be grouped into studies based on whether a particular type of antihypertensive medication was preference, and the loss of sample size after PSM was large, which may have affected the significance of the results and resulted in the failure to identify medications that reduced BPV. Also, we didn't integrate the drug dosage, and the potential impact of alterations in anti-hypertensive regimens or changes in modifiable risk factors may influence the results.

In conclusion, our study of the hypertensive population revealed that the increase in the long-term BPV is an independent risk factor for renal prognosis in hypertensive patients, and the use of RASi can increase the incidence of renal endpoint events but is not related to their resulting increase in the BPV. Therefore, whether RASi are preferred for blood pressure lowering in an older population with a higher BPV deserves further investigation.

## Supplementary Material

Supplementary figures and tables.

## Figures and Tables

**Table 1:** Baseline characteristics of study patients according to BPV quartiles.

Characteristic	Q1 (≤ 0.0432)	Q2 (0.0432 -0.0711)	Q3 (0.0711 -0.1072)	Q4 (> 0.1072)	Total	*P*
	n = 1462	n = 1463	n = 1461	n = 1463	n = 5849	
Age (years)	65(59,73)	65 (60,73)	65(60,73)	66 (60,74)	65 (60, 73)	0.246
Female	440 (30.1%)	439 (30.0%)	461 (31.6%)	585 (40.0%)	1925 (32.9%)	<0.001
Intensive Treatment	673 (46.0%)	713 (48.7%)	753 (51.5%)	780 (53.3%)	2919 (49.9%)	<0.001
Framingham 10-yr CVD risk score	17 (16,19)	17 (16,19)	17 (16,19)	17 (16,19)	17 (16, 19)	<0.001
CVD	243 (16.6%)	254 (17.4%)	285 (19.5%)	293 (20.0%)	1075 (18.4%)	0.048
Framingham 10-yr CVD risk score ≥15%	1391 (95.1%)	1385 (94.7%)	1399 (95.8%)	1388 (94.9%)	5563 (95.1%)	0.567
Current smoker	194 (13.3%)	194 (13.3%)	224 (15.3%)	268 (18.3%)	880 (15.1%)	<0.001
Baseline SBP (mmHg)	137.(130,147)	138 (129,149)	139 (130,149)	140 (130,152)	139 (130, 149)	<0.001
Baseline DBP (mmHg)	79 (71.25,87)	79 (71,86)	80 (72,87)	79 (71,88)	79 (71, 87)	0.352
Heart rate (/min)	66 (59,74)	65 (59,73)	65 (59,73)	65 (57,74)	65 (58, 74)	0.295
BMI (kg/m²)	29.2 (26.3, 33.0)	29.1 (26.1, 32.6)	29.3 (25.9, 33.3)	29.1 (25.8, 33.0)	29.2 (26.1, 33.0)	0.776
eGFR [ml/(min·1.73m2)]	78.7 (69.7, 89.8)	77.4 (69.6, 88.1)	78.1 (69.3, 89.0)	78.4 (68.9, 89.1)	78.1 (69.3, 89.0)	0.238
mALB (mg/dL)	10 (6, 19)	9 (5, 18)	10 (5, 21)	11 (6, 22)	10 (6, 20)	0.003
mALB/Cr (mg/g)	8.2 (5.3, 15.7)	8.0 (5.3, 16.1)	9.0 (5.5, 16.8)	9.5 (5.9, 18.8)	8.6 (5.5, 17.0)	<0.001
TC (mg/dL)	188 (162.25,214)	188 (164,216)	188 (162,215)	189 (161.25,217)	188 (163, 216)	0.887
TG (mg/dL)	104.50 (76,147)	106 (77,154)	105 (75,149)	105 (76,148.75)	105 (76, 149)	0.549
Glucose (mg/dL)	98 (91,106)	97 (91,105)	98 (91,106)	97 (91,105)	97.50 (91, 105)	0.109
Antihypertensive medications						
RASi	926 (63.3%)	1004 (68.6%)	971 (66.5%)	1052 (71.9%)	3953 (67.6%)	<0.001
β-receptor antagonists	459 (31.4%)	458 (31.3%)	515 (35.2%)	607 (41.5%)	2039 (34.9%)	<0.001
CCBs	645 (44.1%)	588 (40.2%)	593 (40.6%)	542 (37.0%)	2368 (40.5%)	0.002
Diuretics	860 (58.8%)	905 (61.9%)	869 (59.5%)	908 (62.1%)	3542 (60.6%)	0.173
Others	114 (7.8%)	121 (8.3%)	109 (7.5%)	143 (9.8%)	487 (8.3%)	0.112

Note: Continuous variables are presented as median (interquartile range); categorical data are presented as frequencies (percentages). Comparison of quantitative variables among the 4 groups was performed by Kruskal-Wallis H test, and comparison of counts was performed by chi-square test. Abbreviations: BMI, body mass index; CCBs, calcium channel blockers; Cr, creatinine; CVD, cardiovascular disease; DBP, diastolic blood pressure; eGFR, estimated glomerular filtration rate; mALB, microalbumin; Q1 to Q4, lowest to highest quartile; RASi, renin-angiotensin-system inhibitors; SBP, systolic blood pressure; TC, total cholesterol; TG, triglyceride.

**Table 2:** Significant risk factors for BPV.

Risk factors	Multivariate β (95% CI)	*P*
Age (per 1-year greater)	0.005 (0 - 0.009)	0.048
Female sex (yes/no)	-0.181 (-0.244 - 0.118)	<0.001
Intensive treatment (yes/no)	0.093 (0.041 - 0.146)	<0.001
Current smoker (yes/no)	0.186 (0.092 - 0.279)	<0.001
Baseline SBP (per 1-mmHg greater)	0.003 (0.000 - 0.005)	0.025
Baseline DBP (per 1-mmHg greater)	0.004 (0.000 - 0.007)	0.029
RASi (yes/no)	0.109 (0.053 - 0.166)	<0.001
β-receptor antagonists (yes/no)	0.166 (0.107 - 0.226)	<0.001
CCBs (yes/no)	-0.113 (-0.167 - 0.059)	<0.001
Other medications (yes/no)	0.137 (0.043 - 0.231)	0.004

Note: Risk factors associated with BPV were analyzed by multiple linear correlation analysis. Abbreviations: CCBs, calcium channel blockers; DBP, diastolic blood pressure; RASi, renin-angiotensin-system inhibitors; SBP, systolic blood pressure.

**Table 3:** Association of BPV with Renal outcome events.

Renal outcome events	BPV		Q1 (n = 2209) 39 events		Q2 (n = 2209) 26 events	Q3 (n = 2208) 53 events		Q4 (n = 2208) 76 events	
	HR (95% CI) per 1-SD increase	*P*	HR (95% CI)	*P*	HR (95% CI)	HR (95% CI)	*P*	HR (95% CI)	*P*
Unadjusted	1.38 (1.23 -1.54)	<0.001	1.50 (0.91 -2.46)	0.109	1.0 (reference)	2.05 (1.28 -3.27)	0.003	2.95 (1.89 -4.61)	<0.001
Model 1	1.32 (1.18 -1.49)	<0.001	1.56 (0.95 -2.56)	0.080	1.0 (reference)	2.00 (1.25 -3.19)	0.004	2.73 (1.75 -4.26)	<0.001
Model 2	1.27 (1.13 -1.44)	<0.001	1.67 (1.01 -2.76)	0.046	1.0 (reference)	2.04 (1.27 -3.29)	0.003	2.53 (1.60 -4.00)	<0.001
Model 3	1.28 (1.13 -1.44)	<0.001	1.68 (1.01 -2.78)	0.044	1.0 (reference)	2.02 (1.25 -3.26)	0.004	2.53 (1.60 -3.99)	<0.001

Note: Cox proportional hazards models were used to estimate HR and 95% CI. The second quartile (Q2) was selected as the reference (n = 2209). Abbreviations: CI, confidence interval; HR, hazard ratio; Q1 to Q4, lowest to highest quartile; SD, standard deviation. Model 1: age, sex, treatment and current smoker. Model 2: Model 1 + SBP, DBP and urine microalbumin. Model 3: Model 2 + RASi, β-receptor antagonists, CCBs and other medications.

**Table 4:** Association of BPV with AKI adverse events.

AKI adverse events	BPV		Q1 (n = 2209) 11 events		Q2 (n = 2209) 9 events	Q3 (n = 2208) 16 events		Q4 (n = 2208) 16 events	
	HR (95% CI) per 1-SD increase	*P*	HR (95% CI)	*P*	HR (95% CI)	HR (95% CI)	*P*	HR (95% CI)	*P*
Unadjusted	1.29 (1.02 -1.63)	0.034	1.50 (0.91 -2.46)	0.639	1.0 (reference)	1.98 (0.88 -4.49)	0.100	1.87 (0.83 -4.24)	0.132
Model 1	1.27 (1.00 -1.61)	0.054	1.27 (0.53 -3.08)	0.595	1.0 (reference)	1.92 (0.85 -4.36)	0.117	1.83 (0.81 -4.16)	0.148
Model 2	1.24 (0.97 -1.59)	0.082	1.24 (0.51 -3.00)	0.635	1.0 (reference)	1.85 (0.82 -4.20)	0.141	1.66 (0.72 -3.78)	0.232
Model 3	1.24 (0.96 -1.58)	0.094	1.28 (0.53 -3.11)	0.586	1.0 (reference)	1.87 (0.82 -4.25)	0.135	1.65 (0.73 -3.77)	0.235

Note: Cox proportional hazards models were used to estimate HR and 95% CI. The second quartile (Q2) was selected as the reference (n = 2209). Abbreviations: CI, confidence interval; HR, hazard ratio; Q1 to Q4, lowest to highest quartile; SD, standard deviation. Model 1: age, sex, treatment and current smoker. Model 2: Model 1 + SBP, DBP and urine microalbumin. Model 3: Model 2 + RASi, β-receptor antagonists, CCBs and other medications.
