# Supplementary material for: Long-term BPV is an Independent Risk Factor for Renal Prognosis in Hypertensive Patients — a Post-hoc Analysis of the SPRINT Study
Source: Int J Med Sci. 2025 Apr 22;22(10):2298–307. doi: 10.7150/ijms.111843 (PMC12080588; doi:10.7150/ijms.111843)
Supplement: Supplementary file 1 — Supplementary figures and tables. [file ijmsv22p2298s1.pdf]

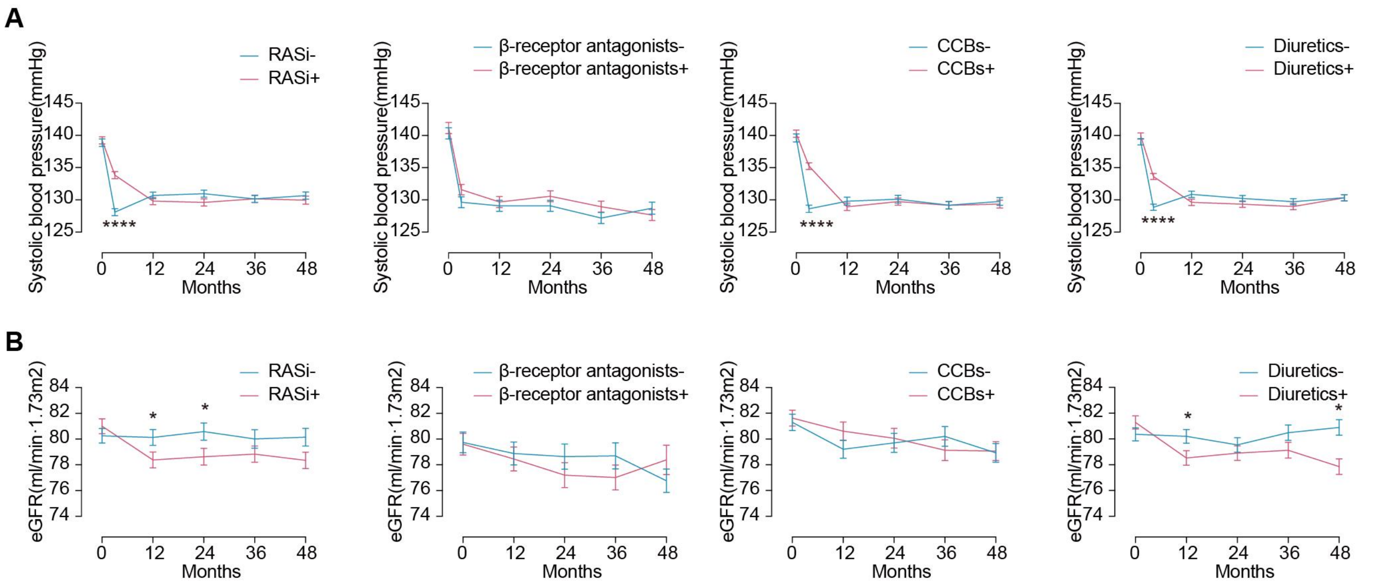

Fig. S1. Systolic blood pressure (**A**) and eGFR (**B**) levels during follow-up of medications preference. Abbreviations: CCBs, calcium channel blockers; eGFR, estimated glomerular filtration rate; RASi, renin-angiotensin-system inhibitors.

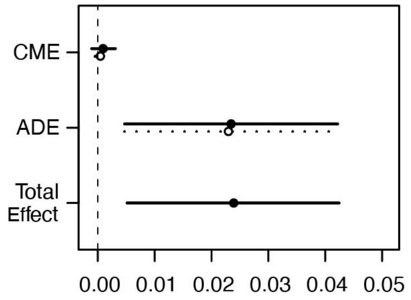

Fig. S2. Mediation analysis of RASi mediated BPV on renal prognosis. Abbreviations: ACME, average causal mediation effects; ADE: average direct effects; BPV, blood pressure variability; RASi, renin-angiotensin-system inhibitors.

Table S1. Baseline characteristics of study patients according to RASi preference.

Characteristic	RASi-	RASi+	Total	*P*
	n = 705	n = 705	n = 1410	
Age (years)	65 (60, 74)	65 (60, 73)	65 (60, 73)	0.801
Female	235 (33.3%)	225 (31.9%)	460 (32.6%)	0.570
Intensive Treatment	512 (36.3%)	250 (35.5%)	262 (37.2%)	0.506
Framingham 10-yr CVD risk score	17 (16, 19)	17 (16, 19)	17 (16, 19)	0.405
CVD	135 (19.2%)	136 (19.3%)	271 (19.2%)	0.946
Framingham 10-yr CVD risk score ≥15%	674 (95.6%)	674 (95.6%)	1348 (95.6%)	1.000
Current smoker	112 (15.9%)	99 (14.0%)	211 (15.0%)	0.332
Baseline SBP (mmHg)	137 (128, 149)	138 (130, 148)	138 (129, 149)	0.421
Baseline DBP (mmHg)	79 (71, 87)	80 (71, 87)	79 (71, 87)	0.783
Heart rate (/min)	65 (58, 74)	66 (59, 73)	65 (58, 73)	0.558
BMI (kg/m^2)	28.9 (26.1, 32.5)	29.5 (26.2, 33.0)	29.2 (26.1, 32.8)	0.136
eGFR [ml/(min·1.73m^2)]	77.0 (68.6, 89.2)	77.7 (69.6, 88.6)	77.5 (69.2, 88.9)	0.320
mALB (mg/dL)	11 (6, 19)	11 (6, 19)	11 (6, 19)	0.614
mALB/Cr (mg/g)	8.2 (5.5, 16.5)	8.4 (5.4, 15.4)	8.3 (5.5, 15.8)	0.485
TC (mg/dL)	189 (164, 218)	187 (163, 213)	188 (163, 215)	0.402
TG (mg/dL)	100 (74, 149)	106 (75, 146)	103 (75, 148)	0.329
Glucose (mg/dL)	97 (91, 105)	98 (91, 106)	98 (91, 105)	0.149

Abbreviations: BMI, body mass index; Cr, creatinine; CVD, cardiovascular disease; DBP, diastolic blood pressure; eGFR, estimated glomerular filtration rate; mALB, microalbumin; RASi, renin-angiotensin-system inhibitors; SBP, systolic blood pressure; TC, total cholesterol; TG, triglyceride.

Table S2. Baseline characteristics of study patients according to β-receptor antagonist preference.

Characteristic	β-receptor antagonist-	β-receptor antagonist+	Total	*P*
	n = 333	n = 334	n = 667	
Age (years)	65 (60, 72)	65 (59, 73)	65 (60, 72)	0.882
Female	124 (37.2%)	117 (35.0%)	241 (36.1%)	0.553
Intensive Treatment	146 (43.8%)	151 (45.2%)	297 (44.5%)	0.723
Framingham 10-yr CVD risk score	17 (16, 19)	17 (16, 19)	17 (16, 19)	0.460
CVD	68 (20.4%)	83 (24.9%)	151 (22.6%)	0.172
Framingham 10-yr CVD risk score ≥15%	311 (93.4%)	309 (92.5%)	620 (93.0%)	0.658
Current smoker	41 (12.3%)	42 (12.6%)	83 (12.4%)	0.918
Baseline SBP (mmHg)	139 (130, 150)	140 (131, 151)	139 (130, 151)	0.407
Baseline DBP (mmHg)	79 (71, 88)	78 (71, 86)	78 (71, 87)	0.204
Heart rate (/min)	62 (55, 72)	61 (56, 70)	61 (55, 71)	0.915
BMI (kg/m^2)	30.1 (27.1, 34.6)	30.1 (27.1, 34.5)	30.1 (27.1, 34.6)	0.861
eGFR [$ml/(min·1.73m^2)$]	76.4 (68.5, 87.3)	76.9 (68.8, 87.5)	76.5 (68.6, 87.4)	0.788
mALB (mg/dL)	10 (6, 20)	12 (6, 21)	11 (6, 20)	0.314
mALB/Cr (mg/g)	8.2 (5.8, 16.1)	9.2 (5.6, 20.4)	8.7 (5.7, 18.3)	0.407
TC (mg/dL)	187 (163, 216)	187 (160, 215)	187 (161, 216)	0.728
TG (mg/dL)	103 (78, 137)	108 (77, 147)	106 (78, 142)	0.392
Glucose (mg/dL)	97 (91, 105)	99 (91, 105)	98 (91, 105)	0.373

Abbreviations: BMI, body mass index; Cr, creatinine; CVD, cardiovascular disease; DBP, diastolic blood pressure; eGFR, estimated glomerular filtration rate; mALB, microalbumin; SBP, systolic blood pressure; TC, total cholesterol; TG, triglyceride.

Table S3. Baseline characteristics of study patients according to CCBs preference.

Characteristic	CCBs -	CCBs+	Total	*P*
	n = 662	n = 663	n = 1323	
Age (years)	65 (60, 74)	65 (60, 73)	65 (60, 74)	0.669
Female	232 (35.1%)	231 (35.0%)	463 (35.0%)	0.970
Intensive Treatment	267 (40.3%)	295 (44.6%)	562 (42.5%)	0.114
Framingham 10-yr CVD risk score	17 (16, 19)	17 (16, 19)	17 (16, 19)	0.582
CVD	112 (16.9%)	118 (17.9%)	230 (17.4%)	0.654
Framingham 10-yr CVD risk score ≥15%	626 (94.6%)	630 (95.3%)	1256 (94.9%)	0.535
Current smoker	96 (14.5%)	90 (13.6%)	186 (14.1%)	0.643
Baseline SBP (mmHg)	138 (129, 149)	139 (130, 149)	139 (129, 149)	0.323
Baseline DBP (mmHg)	79 (71, 87)	79 (71, 87)	79 (71, 87)	0.925
Heart rate (/min)	66 (58, 74)	66 (59, 75)	66 (58, 74)	0.329
BMI (kg/m^2)	29.4 (26.2, 33.3)	29.5 (26.1, 33.5)	29.4 (26.1, 33.4)	0.639
eGFR [ml/(min·1.73m^2)]	78.1 (69.4, 89.1)	78.5 (69.1, 89.9)	78.3 (69.2, 89.3)	0.558
mALB (mg/dL)	10 (6, 20)	13 (7, 25)	11 (6, 23)	<.001
mALB/Cr (mg/g)	8.5 (5.5, 17.2)	9.8 (6.1, 20.0)	9.2 (5.8, 18.4)	0.009
TC (mg/dL)	186 (164, 216)	188 (162, 215)	188 (163, 216)	0.978
TG (mg/dL)	106 (78, 149)	106 (72, 155)	106 (75, 151)	0.624
Glucose (mg/dL)	98 (91, 105)	99 (92, 107)	98 (91, 106)	0.098

Abbreviations: BMI, body mass index; CCBs, calcium channel blockers; Cr, creatinine; CVD, cardiovascular disease; DBP, diastolic blood pressure; eGFR, estimated glomerular filtration rate; mALB, microalbumin; SBP, systolic blood pressure; TC, total cholesterol; TG, triglyceride.

Table S4. Baseline characteristics of study patients according to diuretic preference.

Characteristic	Diuretic-	Diuretic+	Total	*P*
	n = 972	n = 971	n = 1943	
Age (years)	65 (60, 73)	65 (60, 72)	65 (60, 73)	0.685
Female	312 (32.1%)	309 (31.8%)	621 (32.0%)	0.896
Intensive Treatment	399 (41.1%)	404 (41.6%)	803 (41.3%)	0.803
Framingham 10-yr CVD risk score	17 (16, 19)	17 (16, 19)	17 (16, 19)	0.965
CVD	167 (17.2%)	173 (17.8%)	340 (17.5%)	0.712
Framingham 10-yr CVD risk score ≥15%	926 (95.3%)	920 (94.8%)	1846 (95.0%)	0.599
Current smoker	162 (16.7%)	163 (16.8%)	325 (16.7%)	0.943
Baseline SBP (mmHg)	137 (129, 149)	139 (130, 149)	138 (130, 149)	0.103
Baseline DBP (mmHg)	80 (72, 88)	80 (71, 88)	80 (72, 88)	0.888
Heart rate (/min)	65 (58, 75)	66 (59, 74)	66 (59, 75)	0.357
BMI (kg/m^2)	28.9 (26.0, 32.7)	29.4 (26.2, 33.0)	29.1 (26.1, 32.8)	0.187
eGFR [$ml/(min·1.73m^2)$]	77.6 (68.2, 88.4)	77.8 (68.9, 90.3)	77.6 (68.7, 89.0)	0.301
mALB (mg/dL)	11 (6, 19)	11 (6, 21)	11 (6, 21)	0.249
mALB/Cr (mg/g)	8.6 (5.5, 16.6)	9.5 (5.6, 18.7)	9.0 (5.5, 17.6)	0.040
TC (mg/dL)	188 (161, 214)	187 (165, 215)	188 (163, 214)	0.972
TG (mg/dL)	106 (78, 154)	106 (74, 152)	106 (76, 153)	0.227
Glucose (mg/dL)	98 (91, 105)	98 (91, 106)	98 (91, 106)	0.616

Abbreviations: BMI, body mass index; Cr, creatinine; CVD, cardiovascular disease; DBP, diastolic blood pressure; eGFR, estimated glomerular filtration rate; mALB, microalbumin; SBP, systolic blood pressure; TC, total cholesterol; TG, triglyceride.
